# Clinical detection and categorization of uncommon and concomitant mutations involving *BRAF*

**DOI:** 10.1186/s12885-015-1811-y

**Published:** 2015-10-24

**Authors:** Gang Zheng, Li-Hui Tseng, Guoli Chen, Lisa Haley, Peter Illei, Christopher D. Gocke, James R. Eshleman, Ming-Tseh Lin

**Affiliations:** 1 Departments of Pathology, Johns Hopkins University School of Medicine, Baltimore, USA; 2 Department of Medical Genetics, National Taiwan University Hospital, Taipei, Taiwan; 3 Department of Pathology, Penn State Hershey Medical Center, Pennsylvania, USA; 4 Departments of Oncology, Johns Hopkins University School of Medicine, Baltimore, USA

**Keywords:** BRAF, Lung cancer, Colorectal cancer, Melanoma, Next generation sequencing, Kinase activity, Concomitant mutation

## Abstract

**Background:**

Selective BRAF inhibitors, vemurafenib and dabrafenib, and the MEK inhibitor, trametinib, have been approved for treatment of metastatic melanomas with a *BRAF* p.V600E mutation. The clinical significance of non-codon 600 mutations remains unclear, in part, due to variation of kinase activity for different mutants.

**Methods:**

In this study, we categorized *BRAF* mutations according to the reported mutant kinase activity. A total of 1027 lung cancer, colorectal cancer or melanoma specimens were submitted for clinical mutation detection by next generation sequencing.

**Results:**

Non-codon 600 mutations were observed in 37 % of *BRAF*-mutated tumors. Of all *BRAF* mutants, 75 % were kinase-activated, 15 % kinase-impaired and 10 % kinase-unknown. The most common kinase-impaired mutant involves codon 594, specifically, p.D594G (c.1781A > G) and p.D594N (c.1780G > A). Lung cancers showed significantly higher incidences of kinase-impaired or kinase-unknown mutants. Kinase-impaired *BRAF* mutants showed a significant association with concomitant activating *KRAS* or *NRAS* mutations, but not *PIK3CA* mutations, supporting the reported interaction of these mutations.

**Conclusions:**

*BRAF* mutants with impaired or unknown kinase activity as well as concomitant kinase-impaired *BRAF* mutations and *RAS* mutations were detected in lung cancers, colorectal cancers and melanomas. Different therapeutic strategies based on the *BRAF* mutant kinase activity and the concomitant mutations may be worthwhile.

## Background

The mitogen-activated protein kinase (MAPK) or RAS/RAF/MEK/ERK signaling pathway regulates cell proliferation, differentiation and apoptosis [1]. This pathway is often dysregulated in human cancers, frequently due to activating mutations of the *KRAS*, *NRAS*, or *BRAF* genes. Selective BRAF inhibitors like vemurafenib and dabrafenib [2], and MEK inhibitors like trametinib have been developed to target *BRAF* mutant tumors [3]. Since the approval of vemurafenib by the Food and Drug Administration (FDA) of the United States in 2011 for treatment of unresectable or metastatic melanomas with a *BRAF* p.V600E mutation, clinical detection of the *BRAF* p.V600E mutation has become the standard of care for patients with metastatic melanoma in order to predict response to vemurafenib, dabrafenib and trametinib [4–7].

The *BRAF* gene is mutated in approximately 7 % of human cancers overall [8], specifically, 40 % to 60 % of malignant melanomas [9], 10 % to 15 % of colorectal cancers (CRCs) [10], and 1 to 5 % of non-small cell lung cancers (NSCLC) [11, 12]. While p.V600E is the most common mutation detected in many tumor types, more than 100 mutations within exons 11 and 15 of the *BRAF* gene have been reported in the Catalog of Somatic Mutations in Cancer (COSMIC) database, accessed on 03/10/15. The clinical significance of non-codon 600 mutations is largely unknown. In our previous retrospective study for quality assessment, next generation sequencing (NGS) demonstrated a high analytic sensitivity and a broad reportable range for clinical detection of *BRAF* mutations. Non-p.V600E mutants constitute a significant portion of *BRAF* mutations in different tumors: NSCLCs (86 %), melanomas (34 %) and CRCs (23 %) [12]. Discovering the spectrum of non-p.V600E *BRAF* mutations in different malignancies is a first step toward understanding their clinical significance.

**Fig. 1:**
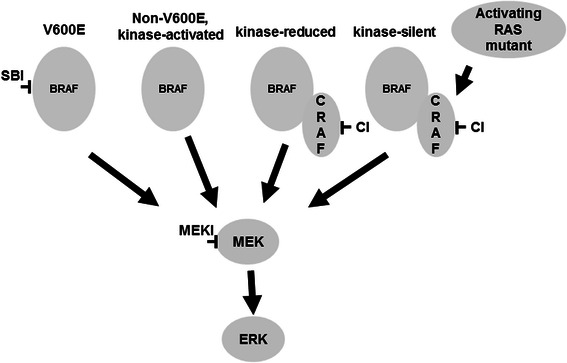
Activation of MAPK pathway by kinase-activated, kinase-impaired and kinase-silent *BRAF* mutants through different mechanisms. Approved or potential targeted therapies are selected based on kinase activity of the *BRAF* mutants and concomitant mutations of the *BRAF* and *RAS* (*KRAS* or *NRAS*) genes. SBI: selective BRAF inhibitors; MEKI: MEK inhibitors; CI: CRAF inhibitors

The role of *BRAF* mutations in the MAPK pathway is complicated not only by the multiplicity of signaling molecular components but also the variation of kinase activity for different *BRAF* mutants (Fig. 1). Most *BRAF* mutations, including the most common p.V600E (c.1799 T > A) mutation, cause upregulation of the kinase activity (kinase-activated mutants). Meanwhile, kinase-impaired *BRAF* mutants have also been reported [11, 13, 14]. While different assay systems have been used to determine the mutant kinase activity, most commonly, basal BRAF kinase activity was determined in vitro by measuring direct MEK phosphorylation [8, 11, 13] or measuring ERK phosphorylation in a kinase cascade assay using purified MEK and ERK proteins [15–17]. Wan et al. defined a high-activity mutant by a basal BRAF kinase activity higher than that of oncogenic RAS-activated wild-type BRAF, an intermediate-activity mutant by a basal BRAF kinase activity between those of wild-type BRAF and oncogenic RAS-activated wild-type BRAF, and a kinase-impaired mutant by a basal BRAF kinase activity lower than that of wild-type BRAF [13]. Demonstration of increased phosphorylation of BRAF and MEK proteins in patient’s tumor cell lysates [18] or inhibition of mutant-induced MEK and/or ERK phosphorylation by BRAF inhibitors in cell culture systems [19, 20] was also used to define kinase-activated mutants. Kinase-impaired mutants were further grouped into reduced-activity mutants, which could still induce MEK and ERK phosphorylation via activation of CRAF in cell culture system (Fig. 1), and silent-activity (or dead) mutant which could not [13, 16, 21]. In the presence of oncogenic RAS, however, the silent-activity mutants could induce MEK and ERK phosphorylation via activation of CRAF (Fig. 1) [22]. Since reduced-activity mutants could still activate MEK/ERK via CRAF [11, 13, 14], demonstration of mutant-induced MEK or ERK phosphorylation in cell culture systems without evidence of inhibition of mutant-induced MEK or ERK phosphorylation by BRAF inhibitors was not sufficient to define a kinase-activated mutant. Kinase-activated mutants and kinase-impaired mutants promote MEK/ERK activation and tumor progression through different mechanisms. Categorization of *BRAF* mutations according to their kinase activity and the presence of absence of concomitant *KRAS* or *NRAS* mutations may shed light on different therapeutic strategies to treat *BRAF*-mutated tumors.

## Methods

### Materials

The Johns Hopkins Medicine institutional review board (IRB) granted approval to this study with waiver of consent. A total of 1027 formalin-fixed paraffin-embedded (FFPE) neoplastic specimens with a diagnosis of lung cancer, colorectal cancer or melanoma were submitted to a *Clinical Laboratory Improvement Amendments* (CLIA)-certified laboratory for mutation detection using a NGS platform between April 2013 and September 2014. NGS failed in 36 (3.5 %) specimens. Fifty-two paired specimens and 3 specimens from one patient showed the same mutation patterns and were counted as one tumor per pair/triad for analysis of the prevalence and spectrum of the *BRAF* mutations. NGS data were available for clinical reporting in 510 lung cancers, 275 CRCs and 152 melanomas. The age of patients ranged from 33–90 (median: 65) for lung cancer specimens, 25–90 (median: 57) for CRC specimens, and 20–94 (median: 61) for melanoma specimens. The proportion of female patients was 55 % for lung cancer specimens, 45 % for CRC specimens, and 36 % for melanoma specimens. Metastatic tumors accounted for 44 % of lung cancer specimens, 28 % of CRC specimens, and 55 % of melanoma specimens. Fifteen melanoma specimens have been tested for a negative p.V600E mutation using pyrosequencing before NGS analysis. Tissue blocks with adequate tumor cellularity were selected by pathologists who made the diagnosis. One Hematoxylin & eosin (H&E) slide followed by 5–10 unstained slides and one additional H&E slide were prepared with PCR precaution. The H&E slides were examined and marked by the pathologist for subsequent macro-dissection of the FFPE neoplastic tissues from 3–10 unstained slides of 5- or 10-micron thick sections. DNA was isolated from the area(s) designated by pathologists using the Pinpoint DNA Isolation System (Zymo Research, Irvine, CA), followed by further purification via the QIAamp DNA Mini Kit (Qiagen, Valencia, CA) [23]. Tumor cellularity was also retrospectively reviewed by two molecular pathologists (GZ and MTL) as 5 quintiles (1–20 %, 21–40 %, 41–60 %, 61–80 % and 81–100 %). In the presence of discrepancy, the mean value was applied.

### Next generation sequencing (NGS)

NGS was conducted using AmpliSeq Cancer Hotspot Panel (v2) for targeted multi-gene amplification as described previously [24, 25]. Briefly, we used Ion AmpliSeq Library Kit 2.0 for library preparation, Ion OneTouch 200 Template Kit v2 DL and Ion OneTouch Instrument for emulsion PCR and template preparation, and Ion PGM 200 Sequencing Kit with Ion 318 Chip and Personal Genome Machine (PGM) as the sequencing platform (Life Technologies, Carlsbad, California), all per manufacturers’ protocol. The DNA input for targeted multi-gene PCR was up to 30 ng measured by Qubit 20 Fluorometer (Life Technologies). Up to 8 specimens were barcoded using Ion Xpress Barcode Adapters (Life Technologies) for each Ion 318 chip. One to three controls (non-template control, a normal peripheral blood control from a male, and/or positive control specimens.) were included in each run. The positive control specimens were prepared from mixture of several cell lines to include mutations in the *AKT*, *BRAF*, *EGFR*, *ERBB2*, *KIT*, *KRAS*, *NRAS* and/or *PIK3CA* genes.

Sequencing data of the targeted genes were analyzed using Torrent Suite (Life Technologies). Lung cancer specimens were tested for *AKT*, *BRAF*, *EGFR*, *ERBB2*, *KRAS*, *NRAS* and *PIK3CA* genes (lung cancer panel), CRC specimens were tested for *BRAF*, *KRAS*, *NRAS* and *PIK3CA* genes (CRC panel), and melanoma specimens were tested for *BRAF*, *KIT*, *NRAS* and *PIK3CA* genes (melanoma panel). *KRAS* mutations were also analyzed for the melanoma specimens. The reference mRNA sequence was NM_005163 for *AKT*, NM_004333 for *BRAF*, NM_033360 for *KRAS*, NM_002524 for *NRAS*, and NM_006218 for *PIK3CA*. Mutations were identified and annotated through both Torrent Variant Caller and direct visual inspection of the binary sequence alignment/map (BAM) file on the Broad Institute’s Integrative Genomics Viewer (IGV) (http://www.broadinstitute.org/igv/). IGV was also used to determine the coverage of each specific exon and to confirm the number of reads of the variants. Novel mutations not reported in the database of COSMIC were confirmed by Sanger sequencing or pyrosequencing as described previously [12]. During our validation of this NGS assay, a cutoff of background noise at 2 % was chosen for single nucleotide variations according to a study of 16 non-neoplastic FFPE tissues [24]. With sufficient DNA input, the limit of detection is dictated by the depth of coverage (or number of sequencing reads). Approximately 150 and 500 reads is needed to detect a heterozygous mutation at a 99 % confidence in a specimen with 20 % and 10 % tumor cellularity, respectively. During the period between April 2013 and September 2014, the coverage of exon 11 and exon 15 of the *BRAF* gene was 1705 ± 1368 and 2182 ± 1540 reads (mean ± standard deviation), respectively.

### Single Nucleotide Polymorphism (SNP) array

SNP array analysis was performed as previously described [26]. Briefly, DNA samples extracted from FFPE tissues (optimally 200 ng) were treated with the Infinium HD FFPE NDA restore kit before running on the Illumina Infinium II SNP array (HumanCytoSNP-12 v2.1 DNA Analysis BeadChip, Illumina Inc., San Diego, CA) according to manufacturer’s standard protocol. The B allele frequency and Log R ratio data were analyzed using Illumina KaryoStudio software version 2.0 and CNV (copy number variation) partition V2.4.4.0.

### Reported mutant kinase activity to categorize *BRAF* mutations observed in the clinical specimens

**Table 1:** Effects of *BRAF* mutations on serine-threonine kinase activity

Activated [references]	Impaired [references]
R462I^a^ [13]	G466E [13, 15]
I463S^a^ [13]	G466R [15]
G464E^a^ [13]	G466V [11, 13]
G464R [15]	G469E^c^ [21]
G464V^a^ [8, 13]	Y472C [11]
G466A^a^ [13]	K483M^b^ [13, 22]
G469A^a^ [8, 11, 13]	D594A^b^ [22]
G469E^a,c^ [13]	D594G^b^ [21]
N581S^a^ [13]	D594V^b^ [13, 16, 22]
E586K [13]	G596R [13, 16]
F595L^a^ [13, 16]	T599A [17]
L597Q [19]	T599I^d^ [16]
L597R [19, 20]	S602A [17]
L597S [19]	
L597V^a^ [8, 11, 13]	
A598V [18]	
T599E [17]	
T599I^a,d^ [13]	
V600D [13]	
V600E [8, 11, 13, 16]	
V600K [13, 15]	
V600R [13, 15]	
K601E [13, 16, 19]	
S602D [17]	
A728V^a^ [13]	

^a^categorized as intermediate activity mutants by Wan et al. [13]

^b^categorized as severely reduced or silent/dead activity mutants

^c^categorized as intermediate activity (only1.8 fold increase) by Wan et al. [13] but reduced activity by Smalley et al. [21]. In the analysis of clinical specimens, p.G469E was grouped into the kinase-unknown category

^d^categorized as intermediate activity by Wan et al. [13], but reduced activity (0.84 fold) by Ikenoue et al. [16]. In the analysis of clinical specimens, p.T599I was grouped into the kinase-unknown category

The majority of mutations are predicted to cause elevation of the kinase activity (Table 1). The degree of elevation varied [13]. Mutations at codon 600 showed several hundred fold elevation of kinase activity while others showed less than 100 fold elevation. Impaired-kinase mutants involving codons 466, 469, 472, 483, 594, 596, 599 and 602 have been reported. The highly conserved aspartic acid residue encoded by codon 594 is a part of the DFG motif that plays an important role in chelating magnesium and stabilizing ATP binding [22]. Mutations at codon 594 of the *BRAF* genes lead to a severely reduced or silent/dead kinase with no direct or indirect activity on the downstream MAPK pathway in the absence of oncogenic RAS [13, 16, 22].

Mutations at the same codon may cause activated or impaired kinase activity depending on the specific mutation. Replacement of the conserved phosphorylation sites at codon 599 and 602 by a non-polar amino acid (such as p.T599A and p.S602A) results in complete abortion of the kinase activity while replacement by an acidic amino acid (such as p.T599E and p.S602D) leads to RAS-independent BRAF activation [17]. A discrepancy has been reported when the codon 599 residue was replaced by a bulky non-polar amino acid, isoleucine (p.T599I mutant) [13, 16]. The p.T599I mutant was categorized as an intermediate-activity mutant by direct measurement of MEK phosphorylation using ATP at a physiological concentration, but showed a slightly deceased basal kinase activity (0.84 fold) by measuring ERK phosphorylation using BRAF kinase cascade assay with ATP at a sub-physiological concentration. Another example occurs at codon 469 of the P loop which is wedged against codon 597 of the activation segment. Replacement by alanine (p.G469A) showed a 200-fold increase of basal kinase activity compared to replacement by bulky glutamic acid (p.G469E) [13]. While p.G469E was categorized as an intermediate-activity mutant, it has the lowest kinase activity within this category (1.8 fold increase) [13]. In contrast, Smalley et al. demonstrated reduced kinase activity of p.G469E mutation [21]. In the following analysis of clinical specimens, p.T599I and p.G469E were therefore grouped into the kinase-unknown category.

### Statistics

Correlation between *BRAF* mutant allele frequency and *KRAS* or *NRAS* mutant allele frequency was examined by Spearman’s rank correlation coefficient (denoted as *r*) using the GraphPad Prism software (GraphPad Software, ver5, La Jolla, CA).

## Results

### Clinical detection of *BRAF* mutations in different tumors according to kinase activity

*BRAF* mutations were detected in 33 of 510 (6.5 %) NSCLCs, 34 of 275 (12 %) CRCs, and 67 of 152 (44 %) melanomas, including a melanoma specimen with both p.V600E (c.1799 T > A) mutation and p.S605I (c.1814G > T) occurring in the same allele (Table 2). The coverage of a *BRAF* mutation was 585 ± 548 reads (mean ± standard deviation). As expected, the most common residue involved by the *BRAF* mutation was codon 600 (86/135, 64 %), followed by codon 594 (15/135, 11 %). Non-codon 600 mutations, p.S467L (c.1400C > T) and p.G594N (c.1780G > A), were detected in 2 of 15 melanomas with prior negative pyrosequencing for codon 600. There was a significant higher fraction of non-codon 600 mutation in NSCLCs (26/33, 79 %) than those in CRCs (7/34, 21 %, *P* < 0.001) and melanomas (16/68, 24 %, *P* < 0.001).

**Table 2:** *BRAF* mutation in lung cancers, CRCs and melanomas

Kinase activity	Mutation	NSCLC	CRC	Melanoma	Total
		(*n* = 510)	(*n* = 275)	(*n* = 152)	(*n* = 937)
Activated					
	G464V (c.1391G > T)	2			2
	G466A (c.1397G > C)	2		1	3
	G469A (c.1406G > C)	1			1
	N581S (c.1742A > G)		2		2
	L597Q (c.1790 T > A)			1	1
	L597R (c.1790 T > G)			1	1
	V600E (c.1799 T > A)^a^	7	27	44	78
	V600K (c.1798_1799delinsAA)			7	7
	V600R (c.1798_1799GT > AG)			1	1
	K601E (c.1801A > G)	3		2	5
	Total	15 (2.9 %)	29 (11 %)	57 (38 %)	101
Impaired					
	G466R (c.1396G > A)	1			1
	G466V (c.1397G > T)	1		1	2
	Y472C (c.1415A > G)		1		1
	D594E (c.1782 T > G)			1	1
	D594G (c.1781A > G)	4	3	1	8
	D594H (c.1780G > C)	1			1
	D594N (c.1780G > A)	2	1	2	5
	G596R (c.1786G > C)	1			1
	Total	10 (2.0 %)	5 (1.8 %)	5 (3.3 %)	20
Unknown					
	T440I (c.1319C > T)^b^	1			1
	S467L (c.1400C > T)			1	1
	G469E (c.1406G > A)			2	2
	G469R (c.1405G > A)	1			1
	G469S (c.1405_1406delinsTC)	1			1
	G469V (c.1406G > T)	1		1	2
	L584F (c.1750C > T)			1	1
	L588F (c.1762C > T)	1			1
	V600_K601delinsE (c.1799_1801del)^b^	1			1
	S605I (c.1814G > T)^b^			1	1
	Q609L (c.1825_1826delinsTT)^b^	1			1
	E611Q (c.1831G > C)^b^	1			1
	Total	8 (1.6 %)	0	6 (3.9 %)	14
Total		33 (6.5 %)	34 (12 %)	68/67^c^ (44 %)	135/134^c^

^a^including a melanoma specimen with c.1799_1800delinsAA (p.V600E2)

^b^mutations not reported in the COSMIC database (last assessment on August 7, 2015)

^c^including a melanoma specimen with both p.V600E and p.S605I mutations

**Fig. 2:**
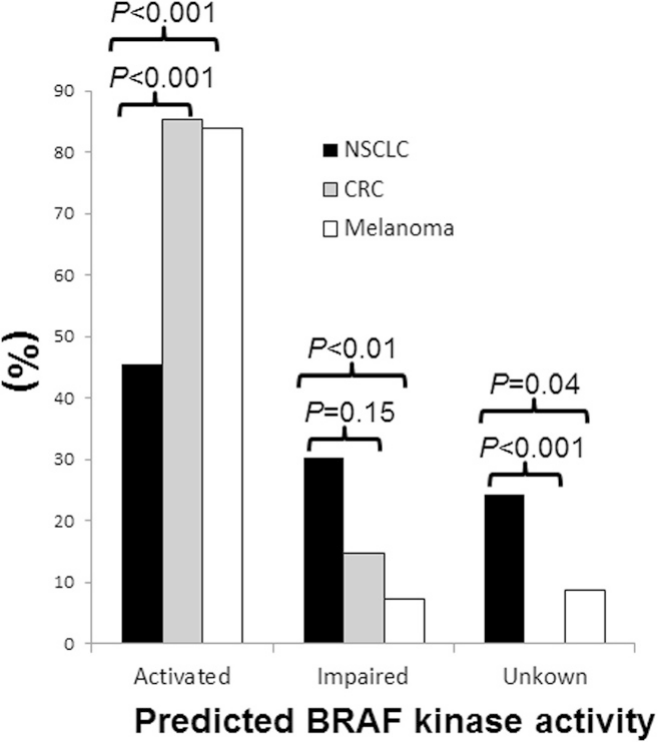
Distribution of kinase-activated, kinase-impaired and kinase-unknown *BRAF* mutants. Non-small cell lung cancers (NSCLCs) showed higher incidences of kinase-impaired and kinase-unknown *BRAF* mutants as compared to colorectal cancers (CRCs) and melanomas

Kinases activity is predicted to be elevated in 101 of 135 (75 %) *BRAF* mutations, impaired in 20 (15 %) and unknown in 14 (10 %). Unique mutations detected in only one tumor were observed in 4 of 10 unique kinase-activated mutants, 4 of 8 kinase-impaired mutants and 10 of 12 kinase-unknown mutants. The exceptions for the kinase-unknown mutant were p.G469E (c.1406G > A) and p.G469V (c.1406G > T). The most common residue involved by the kinase-impaired mutant was codon 594 (15 of 20 or 75 % of kinase-impaired mutants), specifically, p.D594G (c.1781A > G) in 8 tumors and p.D594N (c.1780G > A) in 5 tumors. Mutations involving codon 594 were observed in 7 (1.4 %) of 510 lung cancers, 4 (1.5 %) of 275 CRCs sand 4 (2.6 %) of 152 melanomas. Other kinase-impaired mutants included p. G466R (c.1396G > A), p.G466V (c.1397G > T), p.Y472C (c.1415A > G) and p.G596R (c.1786G > C). Six of 14 kinase-unknown mutants were seen in codon 469. Among those tumors with a *BRAF* mutation, NSCLCs showed a significant lower incidence of kinase-activated mutants (45 %) as compared to CRCs (85 %) and melanomas (84 %) and a higher incidence of kinase-impaired mutants or kinase-unknown mutants (Fig. 2). *BRAF* mutations with unknown kinase activity were not seen in CRC specimens.

### Concomitant mutations of MAPK pathway

**Fig. 3:**
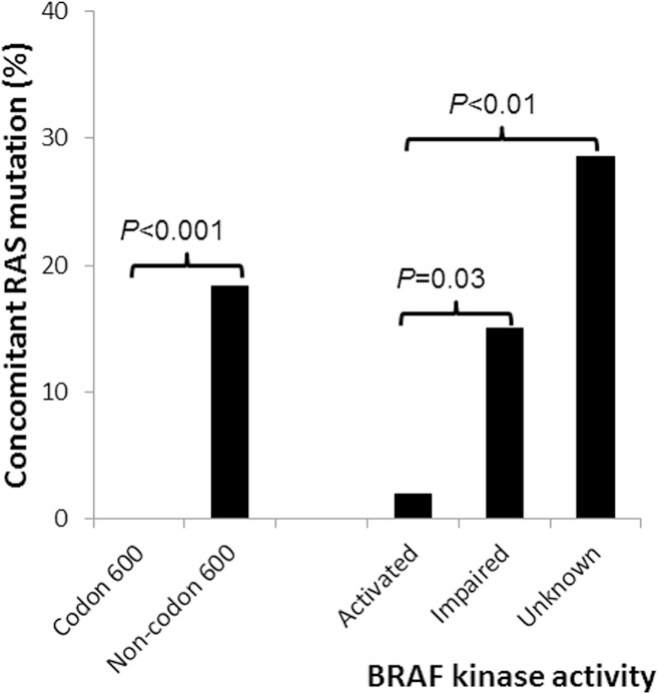
Incidence of concomitant *BRAF* and activated *RAS* (*KRAS* or *NRAS*) mutations. Higher incidences of concomitant *BRAF* and activated *RAS* mutations are seen in non-codon 600 *BRAF*-mutated tumors (left) and in BRAF-mutated tumors with impaired or unknown kinase activity (right)

No concomitant *BRAF* and *EGFR* or *ERBB2* mutation was observed in NSCLC specimens. The frequency of concomitant *KRAS* or *NRAS* mutations found in a *BRAF*-mutated tumor was 4 of 33 (12 %) in NSCLCs, 3 of 34 (8.8 %) in CRCs, or 3 of 67 (4.5 %) in melanomas (Table 3). An NSCLC specimen showed an additional *KRAS* p.V8I (c.22G > A) mutation in the same allele of p.G13D (c.38G > A) mutation (case P10) and a melanoma specimen showed an addition *PIK3CA* p.P75S (c.169C > T) mutation (case P8). All except *KRAS* p.G15S (c.43G > A) in case P2 were activating *KRAS* or *NRAS* mutations at codon 12, 13, 59 or 61. Concomitant *BRAF* and activating *RAS* mutations were observed in 0 of 86 specimens with codon 600 mutations and in 9 of 49 specimens (18 %) with non-codon 600 mutations (Fig. 3). Concomitant *BRAF* and activating *RAS* mutations were observed in 2 of 101 (2.0 %) kinase-activated mutants, 3 of 20 (15 %) kinase-impaired mutants, and 4 of 14 (29 %) kinase-unknown mutants (Fig. 3).

**Table 3:** Concomitant *BRAF* mutations with *KRAS* or *NRAS* mutations in the MAPK pathway

Kinase activity	Diagnosis^a^	*BRAF* ^b^	*RAS* ^c^
Activated			
Case P1	Melanoma (71–90 %)	G466A^d^ (30 %)	*KRAS*/G12D (49 %)
Case P2	colorectal cancer (41–60 %)	V600E (29 %)	*KRAS*/G15S (8.4 %)
Case P3	Melanoma (61–80 %)	K601E (35 %)	*NRAS*/G13N (34 %)
Impaired			
Case P4	Colorectal cancer (71–90 %)	Y472C (40 %)	*KRAS*/G12V (40 %)
Case P5	Colorectal cancer (51–70 %)	D594G (31 %)	*KRAS*/A59E (30 %)
Case P6	Lung cancer (51–70 %)	D594N (5.7 %)	*KRAS*/Q61H (24 %)
Unknown			
Case P7	Lung cancer (41–60 %)	T440I (13 %)	*KRAS*/G12V (16 %)
Case P8^e^	Melanoma (61–80 %)	S467L (26 %)	*NRAS*/Q61K (24 %)
Case P9	Lung cancer (21–40 %)	G469R (11 %)	*KRAS*/G12R (12 %)
Case P10	Lung cancer (41–60 %)	G469V (20 %)	*KRAS*/G13D (25 %)

^a^Estimated tumor cell percentage of the specimens was indicated in the parenthesis

^b^Nucleotide changes of *BRAF* mutations were shown in Table 2. Percentage in the parenthesis indicates mutant allele frequency

^c^activating *KRAS* or *NRAS* mutations except *KRAS* p.G15S (c.43G > A) of unknown significance. G12V: c.35G > T;; G12R: c.34G > C; G13D: c.38G > A; A59E: c.176C > A; Q61H: c.183A > C; Q61K: c.181C > A. Percentage in the parenthesis indicates mutant allele frequency

^d^intermediate -activity mutant by Wan et al. [13]

^e^same case as M8 with *BRAF*, *NRAS* and *PIK3CA* mutations in Table 4

**Fig. 4:**
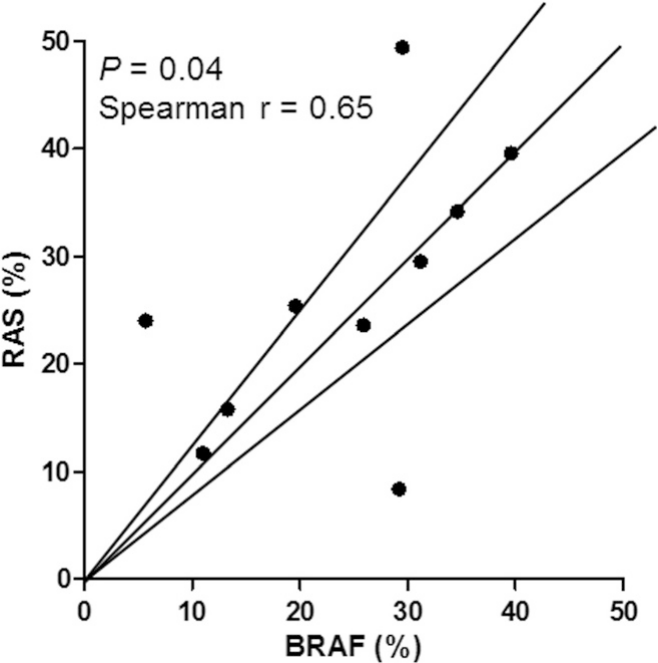
Correlation of mutant allele frequencies in tumors with concomitant *BRAF* and activating *RAS* mutations. The lines labeled with 80 % and 120 % indicate the boundary of events with the *BRAF*/*RAS* mutant allele ratio between 80 % and 120 %. *r*: Spearman’s rank correlation coefficient

*BRAF* mutant allele frequencies were highly concordant with the *KRAS* and *NRAS* mutant allele frequencies (Fig. 4), suggesting that concomitant mutations are present in the same tumor population. A discrepancy was observed in cases P1 with a higher *KRAS* mutant allele frequency (49 % vs. 30 %), case 2 with a much lower *KRAS* p.G15S (c.43G > A) allele frequency (8.4 % vs. 29 %), and case P6 with a much lower *BRAF* p.D594N (c.1780G > A) allele frequency (5.7 % vs. 24 %). Pyrosequencing was performed in DNA specimens isolated from 3 subareas of case P1. The *KRAS*/ *BRAF* mutant allele ratio was 2.04 (49 % vs. 24 %), 1.72 (50 % vs. 29 %) and 1.59 (53 % and 34 %), respectively. SNP array analysis of case P1 revealed gain of chromosome 12p containing the *KRAS* gene. These results indicate that concomitant *KRAS* and *BRAF* mutations are present within the same tumor cells with amplification of the *KRAS* mutant allele. In case P2, the *BRAF* p.V600E (c.1799 T > A) mutant allele frequency (29 %) was consistent with the estimated tumor cellularity (41–60 %), suggesting *KRAS* p.G15S (c.43G > A) was present in a subpopulation of tumor. This was confirmed by the presence of *BRAF* p.V600E (c.1799 T > A) mutation in all 4 subareas, but *KRAS* p.G15S (c.43G > A) mutation (16 % vs. 34 % of *BRAF* p.V600E) in only one of 4 subareas. SNP array showed no aneuploidy of both chromosomes 12 and 7 containing *KRAS* gene and *BRAF* gene, respectively. Similarly in case P6, the *BRAF* p.D594N (c.1780G > A) mutant was also likely present in a subpopulation of tumors.

The presence of 40 % *BRAF* and *KRAS* mutations in the context of 71–90 % estimated tumor cellularity in case P4 and the presence of 31 % *BRAF* mutation and 30 % *KRAS* mutation in the context of 51–70 % estimated tumor cellularity in case P5 suggest that the kinase-impaired *BRAF* mutation and the activating *KRAS* mutation were present in the all the tumor cells. *KRAS*/*BRAF* mutant allele ratio was consistently 1:1 (1.05, 1.06 and 1.00 by pyrosequencing) in 3 subareas re-isolated from cases P5, further supporting the presence of concomitant mutations in the same tumor cell population instead of different tumor subpopulations.

### Concomitant mutations of mTOR pathway

Concomitant *BRAF* and *AKT* mutations were observed in 2 lung adenocarcinomas (Table 4), both of which had a *BRAF* p.V600E (c.1799 T > A) mutation. *BRAF* mutations accompanied by a *PIK3CA* mutation were observed in 2 of 33 (6.1 %) *BRAF*-mutated lung cancers, 4 of 34 (12 %) *BRAF*-mutated CRCs, and 2 of 67 (3.0 %) *BRAF*-mutated melanomas (Table 4). Concomitant *BRAF* and *PIK3CA* mutations were observed in 4 of 86 specimens (4.7 %) with a codon 600 mutation and in 4 of 49 specimens (8.2 %) with a non-codon 600 mutation (*P* = 0.46 by Fisher exact test). Concomitant *PIK3CA* mutations were observed in 4 of 101 (4.0 %) elevated-activity *BRAF* mutants, 1 of 20 (5.0 %) reduced-activity mutants, and 3 of 14 (21 %) unknown-activity mutants.

**Table 4:** Concomitant *BRAF* mutations with *AKT* or *PIK3CA* mutations in the mTOR pathway

Kinase activity	Diagnosis	*BRAF* ^a^	*AKT* ^b^ */PIK3CA* ^*c*^
Activated			
Case M1	lung cancer	V600E (13 %)	*AKT*/E17K (13 %)
Case M2	lung cancer	V600E (24 %)	*AKT*/E17K (30 %)
Case M3	lung cancer	V600E (6.2 %)	*PIK3CA*/R88Q (5.7 %)
Case M4	colorectal cancer	V600E (24 %)	*PIK3CA*/E545K (29 %)
Case M5	colorectal cancer	V600E (19 %)	*PIK3CA*/H1047Q (25 %)
Case M6	colorectal cancer	V600E (42 %)	*PIK3CA*/H1047R (39 %)
Impaired			
Case M7	melanoma	D594N (59 %)	*PIK3CA*/L327F (19 %)
Unknown			
Case M8^d^	melanoma	S467L (26 %)	*PIK3CA*/P57S (23 %)
Case M9	colorectal cancer	N581S (30 %)	*PIK3CA*/K111E (32 %)
Case M10	lung cancer	E611Q (18 %)	*PIK3CA*/D350N (13 %)

Percentage in the parenthesis indicates mutant allele frequency

^a^Nucleotide changes of *BRAF* mutations were shown in Table 2

^b^E17K: c.49G > A

^c^P57S: c.169C > T; p.R88Q (c.263G > A); p.K111E (c.331A > G); L327F: c.979C > T; D350N: c.1048G > T; p.E545K: c.1633G > A; p.H1047Q (c.3141 T > G); p.H1047R: c.3140A > G

^d^same case as P8 with *BRAF*, *NRAS* and *PIK3CA* mutations in Table 3

## Discussion

*BRAF* mutations show diverse functional consequences and varied response to BRAF inhibitors. In this study, we categorized *BRAF* mutations detected in NSCLCs, CRCs and melanomas into kinase-activated mutants (75 %), kinase-impaired mutants (15 %) and kinase-unknown mutants (10 %) according to the functional studies reported in the literature. NSCLCs showed a significantly lower incidence of kinase-activated mutants than those of CRCs and melanomas. Mutations at codon 594 accounted for 11 % of *BRAF* mutations and were the most common kinase-impaired ones. We also demonstrated that concomitant *KRAS* or *NRAS* mutations, but not *PIK3CA* mutations, more likely occur with the kinase-impaired *BRAF* mutants than the kinase-activated ones.

While some *BRAF* mutations could be passenger mutations, especially those with impaired or unknown kinase activity, most *BRAF* mutations with elevated kinase activity are likely involved in oncogenesis and thus could be targetable. Choices of the inhibitors or inhibitor combinations are at least partly made based on *BRAF* mutation status. In our study, p.V600E (c.1799 T > A) was the most common *BRAF* mutation, occurring in 27 of 34 (79 %) CRCs, 44 of 68 (65 %) melanomas and 7 of 33 (21 %) NSCLCs. Non-p.V600E codon 600 mutations were seen in 8 of 68 (12 %) *BRAF*-mutated melanomas. The selective inhibitors of *BRAF* codon 600 mutants, vemurafenib and dabrafenib, have been shown to improve progression-free and overall survival in metastatic melanoma patients, with either the p.V600E mutation or non-p.V600E mutations at codon 600, such as p.V600K and p.V600R [4, 27–29]. Responding to vemurafenib or dabrafenib has been observed in few NSCLC patients with a *BRAF* p.V600E mutation [30–33], although the exact benefit of selective BRAF inhibitors for lung cancer patients is currently still under investigation [34]. Whether BRAF inhibitors benefit patients with a kinase-activated *BRAF* mutation located outside codon 600 remains less clear, although a partial response to vemurafenib has been reported in a melanoma patient with a p.L597R mutation [20]. Responsiveness to MEK inhibitors (TAK-733 and trametinib) in melanoma patients with a codon 597 mutations or p.K601E mutation suggests that future clinical trials of MEK inhibitors in patients with kinase-activated non-codon 600 mutations should be considered [19, 35, 36].

*BRAF* mutations with reduced kinase activity are unlikely responsive to BRAF inhibitors. These mutants, however, can still drive MAPK pathway through activation of CRAF/MEK/ERK cascade (Fig. 1) [13, 14]. In vitro studies of kinase-reduced mutants such as p.G466V, p.G466E and p.G596R, have also shown that activation of the CRAF/MEK/ERK cascade can be inhibited by MEK inhibitors or sorafenib, an inhibitor for multiple kinase including CRAF. In a previous clinical trial of dasatinib, a tyrosine kinase inhibitor, for metastatic non-small cell lung cancer, a patient with *BRAF* p.Y472C mutation remained 4-year disease-free after treatment [11]. In vitro studies confirmed the activation of CRAF/MEK/ERK cascade by the kinase-impaired p.Y472C mutant and demonstrated dasatinib-induced senescence and apoptosis in lung cancer cells expressing kinase-impaired p.G466V mutant, but not in cell lines with kinase-activated *BRAF* mutants.

Mutations with silent/dead kinase activity are unlikely responsive to either BRAF or MEK inhibitors. Mutations with silent/dead kinase activity have been reported in p.D594V, p.D594A and p.K483M mutations of the *BRAF* gene [13, 16, 22]. Codon 483 encodes the catalytic lysine and the aspartic acid at codon 594 is part of the DFG motif that plays an important role in chelating magnesium and stabilizing ATP binding [22]. Mutations at codon 483 or 594 genes lead to silence of the kinase activity with no direct or indirect activation on the downstream MAPK pathway [13, 16, 22]. Mutations at codon 594, however, have been associated with a higher incidence of co-existent *RAS* mutation (4 in 34, 12 %) as compared to p.V600E and presumably may also cooperate with mutations within the upstream of RAS or inter-connected pathways [22]. In this study, mutations at codon 594 constituted the second most common *BRAF* mutations in NSCLCs (7/33, 21 %), CRCs (4/34, 12 %) and melanomas (4/68, 5.9 %). Concomitant activating *RAS* mutations were observed in 2 of 15 (13 %) codon 594 mutations, but none of 86 codon 600 mutations. In the presence of oncogenic RAS proteins, kinase-silent BRAF forms a complex with CRAF and lead to hyperactivation of the CRAF/MEK/ERK cascade (Fig. 1) [22]. These preclinical studies suggested that MEK inhibitors or CRAF inhibitors may benefit patients with concomitant kinase-silent *BRAF* mutation and activating *RAS* mutation.

In contrast to the absence of concomitant *BRAF* p.V600E mutation and activating *RAS* mutation, 3 of 7 (43 %) NSCLCs and 3 of 27 (11 %) CRCs with a p.V600E (c.1799 T > A) showed a concomitant mutation in the *AKT* or *PIK3CA* genes of the mTOR pathway. In the step-wise genetic alteration model associated with colorectal tumorigenesis, *PIK3CA* mutations occur after *KRAS* or *BRAF* mutations and, in cooperation with other mutations, drive clonal evolution from large adenoma to invasive adenocarcinoma [37]. Thus it is not surprising to see concomitant *PIK3CA* mutations in 11 % of CRCs with a *BRAF* p.V600E (c.1799 T > A) mutation, similar to a 16 % of CRCs with an activating *KRAS* mutation (data not shown) in this cohort. Activating *PIK3CA* p.H1047R mutation has also been shown to cooperate with *BRAF* p.V600E mutation to promote progression of benign lung tumors to lung cancers [38]. Mutations in the *AKT* and *PIK3CA* genes, however, are uncommon in NSCLCs. *PIK3CA* mutations have been detected in approximately 2 % of lung adenocarcinomas [39]. The *AKT* p.E17K mutation was seen in only 2 of 509 NSCLCs [40–43]. In this study, the *AKT* p.E17K mutation was only detected in two lung adenocarcinomas with a *BRAF* p.V600E (c.1799 T > A) mutation, suggesting the cooperation between the MAPK and mTOR pathways, similar to that between the *KRAS*, *NRAS* or *BRAF* mutation and *PIK3CA* mutation.

In general, initiating driving mutations within the same pathway are mutually exclusive. In the setting of clinical diagnosis, caution has to be taken for interpretations of “double initiating mutations” within the same pathway. Mutations in the *KRAS*, *NRAS* and *PIK3CA* genes have been the mechanisms for both innate and acquired resistance to targeted therapeutics with kinase inhibitors or anti-EGFR antibodies [34]. In this study, none of patients with concomitant mutations received kinase inhibitors or anti-EGFR antibodies. In the presence of concomitant *PIK3CA* or *AKT* mutations, a combination of BRAF inhibitors or MEK inhibitors with mTOR pathway inhibitors may be more effective [44]. Correlation of the mutant allele frequencies with the estimated tumor frequency may be applied to elucidate if concomitant mutations are present in the same tumor population or only in a subpopulation. The consistency of mutant allele ratios, when testing different random subareas, would further support that concomitant mutations are present in the same population.

## Conclusion

In this study, we categorized *BRAF* mutations according to the reported kinase activity and showed that concomitant *KRAS* or *NRAS* mutations more likely occur with the kinase-impaired *BRAF* mutants than the kinase-activated *BRAF* mutants. Different therapeutic strategies should be developed based on *BRAF* mutant kinase activity and the concomitant mutations.

## Abbreviations

BRAF: v-raf murine sarcoma viral oncogene homologe B1
COSMIC: Catalog of Somatic Mutations in Cancer
CRAF: v-raf-1 murine leukemia viral oncogene homolog 1
CRC: Colorectal cancer
EGFR: Epidermal growth factor receptor
ERBB2: v-erb-b2 avian erythroblastic leukemia viral oncogene homolog 2
ERK: Extracellular-signal-regulated kinase
FFPE: Formalin-fixed paraffin-embedded
IGV: Integrative genomics viewer
KRAS: V-Ki-ras2 Kirsten rat sarcoma viral oncogene homolog
MAPK: Mitogen-activated protein kinase
MEK: Mitogen-activated protein kinase kinase
mTOR: Mammalian target of rapamycin
NGS: Next generation sequencing
NRAS: Neuroblastoma RAS viral oncogene homolog
NSCLC: Non-small cell lung cancer
PIK3CA: Phosphoinositide-3-kinase-catalytic-alpha
